# Designation of a Novel DKK1 Multiepitope DNA Vaccine and Inhibition of Bone Loss in Collagen-Induced Arthritic Mice

**DOI:** 10.1155/2015/765490

**Published:** 2015-05-05

**Authors:** Xiaoqing Zhang, Sibo Liu, Shentao Li, Yuxuan Du, Yunpeng Dou, Zhanguo Li, Huihui Yuan, Wenming Zhao

**Affiliations:** ^1^Department of Immunology, School of Basic Medical Sciences, Capital Medical University, No. 10 Xitoutiao, You An Men, Fengtai, Beijing 100069, China; ^2^Department of Immunology, College of Medical Sciences, China Medical University, No. 77 Puhe Road, Shenyang North Area, Shenyang, Liaoning 110122, China; ^3^Department of Rheumatology & Immunology, Clinical Immunology Center, Peking University People's Hospital, No. 11 Xizhimen South Street, Beijing 100044, China

## Abstract

Dickkopf-1 (DKK1), a secretory inhibitor of canonical Wnt signaling, plays a critical role in certain bone loss diseases. Studies have shown that serum levels of DKK1 are significantly higher in rheumatoid arthritis (RA) patients and are correlated with the severity of the disease, which indicates the possibility that bone erosion in RA may be inhibited by neutralizing the biological activity of DKK1. In this study, we selected a panel of twelve peptides using the software DNASTAR 7.1 and screened high affinity and immunogenicity epitopes *in vitro* and *in vivo* assays. Furthermore, we optimized four B cell epitopes to design a novel DKK1 multiepitope DNA vaccine and evaluated its bone protective effects in collagen-induced arthritis (CIA), a mouse model of RA. High level expression of the designed vaccine was measured in supernatant of COS7 cells. In addition, intramuscular immunization of BALB/c mice with this vaccine was also highly expressed and sufficient to induce the production of long-term IgG, which neutralized natural DKK1 *in vivo*. Importantly, this vaccine significantly attenuated bone erosion in CIA mice compared with positive control mice. These results provide evidence for the development of a DNA vaccine targeted against DKK1 to attenuate bone erosion.

## 1. Introduction

Rheumatoid arthritis (RA), a chronic symmetrical autoimmune disease, is characterized by synovial inflammation and proliferation accompanied by cartilage erosion and bone loss [1]. More than one-third of patients eventually experience employment disability and lower quality of life because of this disease, which is largely responsible for the high socioeconomic burden of RA [2]. Furthermore, mortality rates in RA patients are higher than in the healthy population [3].

The aim of RA treatment is the achievement of remission [4], but many RA patients who are judged by their consulting rheumatologist to be in remission after receiving conventional therapy still show structural deterioration [5]. Therefore, the long-term goals of treatment are to prevent joint destruction and the comorbidities of the disease [3]. A number of reports have suggested that modern therapy does not inhibit joint damage satisfactorily despite achieving clinical remission [6]. Therefore, the development of effective agents to inhibit bone erosion in RA patients is urgent.

The canonical Wnt signaling pathway promotes bone formation not only by stimulating the differentiation of osteoblasts, increasing the growth rate of osteoblasts and reducing their apoptosis but also by inhibiting osteoclastogenesis [7]. The canonical Wnt signaling pathway is triggered by the association of secretory Wnt with its frizzled (Fz) receptors and low density lipoprotein receptor-related protein 5/6 (LRP5/6) on the cell surface. This stabilizes *β*-catenin, which is eventually translocated into the nucleus, and activates the TCF/LEF-mediated transcription of target genes that elicit a variety of effects, including the induction of osteoblast differentiation and proliferation [8]. Dickkopf-1 (DKK1), a soluble and natural inhibitor of the canonical Wnt signaling pathway, may play an active role in inhibiting osteogenesis by binding the ligands of Wnt proteins [9, 10]. In addition to LPR5/6, DKK1 was also found to bind to Kremen, another cell surface coreceptor, forming a ternary complex that is rapidly endocytosed, resulting in the depletion of cell surface LRP5/6 [11]. Studies have demonstrated that the levels of DKK1 in serum were significantly higher in RA patients and were correlated with the severity of the disease [12]. In a mouse model of RA, treatment with an anti-DKK1 antibody has attenuated bone erosion [9]. Therefore, DKK1 may be a promising therapeutic target for RA bone loss.

Over the last 20 years, great progress has been made in developing DNA vaccines [13]. DNA vaccines have many advantages over conventional chemical agents, biological agents, and protein vaccines in the treatment of diseases. First, plasmid preparation is rapid and cost effective and does not suffer from problems such as improper protein folding. Plasmid DNA is highly stable and flexible, allowing for the modification of plasmid sequences [14]. In addition, the antigen presenting cells (APCs) process and present the epitopes from antigens on MHC I and II molecules, thereby inducing both humoral immunity and cellular immunity. Active immunotherapy is feasible to reverse the disorders of autoimmune diseases. DNA vaccines have proven effective in animal models, including RA, Crohn's disease, systemic erythematosus lupus (SLE), and infectious diseases [15] and have been extensively evaluated in humans. The advancements of bioinformation software and molecular immunology promoted the development of DNA vaccine [16]. Currently, 72 Phase I, 20 Phase II, and 2 Phase III clinical trials have been identified [17].

In this study, we selected a panel of twelve peptides using the software DNASTAR 7.1 and screened high affinity and immunogenicity epitopes* in vitro* and* in vivo* assays. Then, we optimized four B cell epitopes and designed a novel DKK1 multiepitope DNA vaccine, determined its immunogenicity, and evaluated its protective effects. Our data demonstrated that this DNA vaccine ameliorated bone erosion significantly in mice with collagen-induced arthritis (CIA).

## 2. Materials and Methods

### 2.1. Cells and Mice

COS7 cells were grown in Dulbecco's modified Eagle's medium (DMEM) supplemented with 5% fetal bovine serum (FBS) in a humidified 5% CO_2_ incubator at 37°C.

Six-week-old female BALB/c (H-2d) mice and 5-week-old male DBA/1 mice (a CIA-susceptible mouse strain, H-2q) were both purchased from HFK Biotechnology Co. Ltd. All mice were maintained in a specific pathogen-free environment. All animal experiments were performed according to the guidelines of the Animal Care and Use Committee of Capital Medical University.

### 2.2. Construction and Preparation of the DNA Vaccine

B cell epitopes in the amino acid sequence of human DKK1 were analyzed using the software DNASTAR 7.1. The separated epitopes were synthesis from Invitrogen (Life Technologies, California, USA). Subsequently, the indirect ELISA was utilized to detect the affinity of separated epitopes. Simply, 96-well plates were coated with peptides (1 *μ*g/mL) overnight at 4°C. The DKK1 polyclonal antibodies (diluted 1 : 1000, 200 *μ*L, R&D Systems, Minneapolis, MN, USA) were added to wells and incubated overnight at 4°C. After washing, the HRP conjugate goat anti-human DKK1 secondary antibody (100 *μ*L, diluted 1 : 2000, Southern Biotech, Birmingham, AL, USA) was added and the plates were incubated for 1 h at 37°C. Then, the peptides were immunized BALB/c mice and the immunogenicity was measured as previously described [18]. The reactions were stopped by the addition of 50 *μ*L of Stop Solution (R&D Systems), and OD450 readings were measured with an ELISA plate reader. To reduce the bioactivity of DKK1 vaccine, the peptides (100 *μ*g/injection, intraperitoneally) were applied for a week. The tibias were collected and an acid phosphatase kit (Sigma-Aldrich, St. Louis, MO, USA) was used to demonstrate osteoclasts [19].

Four fragments in the DKK1 sequence (110–144aa, 153–181aa, 182–216aa, and 228–253aa) with high affinity and immunogenicity were selected to construct the multiepitope DNA vaccine. The DNA sequences encoding four amino acids (R203, H204, K211, and R236) in DKK1 were mutated to glutamate to reduce the biological activity of DKK1 [20]. AAY spacers between two adjacent epitope fragments were used to link the four selected epitopes. A Th2 cell epitope was added at both sequence termini. Finally, the signal peptide of human DKK1 was added at the N-terminus of the sequence. The synthetic nucleotide sequence, named DP, was incorporated into the expression vector pCMV6-XL5 using a standard DNA recombination procedure, resulting in the recombinant plasmid pCMV-DP. Plasmids for immunization were extracted and purified from transformed* Escherichia coli* strain DH5*α* using an endotoxin-free plasmid extraction kit (Roche, Mannheim, Germany) according to the manufacturer's instructions. The purified plasmids were adjusted to a concentration of 1 mg/mL in sterile saline and stored at −80°C.

### 2.3. Transfection of Plasmid pCMV-DP into COS7 Cells

COS7 cells were cultured in a 6-well tissue culture plate until the cells reached approximately 60% to 80% confluence. The cells were transfected with the purified plasmid DNA using TurboFect* in vitro *Transfection Reagent (Fermentas, ThermoScientific, Pittsburgh PA, USA) according to the manufacturer's instructions. Briefly, 4 *μ*L of plasmid DNA (1 mg/mL) was diluted in 390 *μ*L of serum-free DMEM. TurboFect reagents (6 *μ*L) were added to the diluted DNA. After immediate mixing and incubation for 20 min at room temperature, 400 *μ*L of the mixture was distributed dropwise into cultured COS7 cells in a 6-well plate. The transfected cells were cultured for 72 h. Cell lysates and culture supernatants were collected for further analyses.

### 2.4. Western Blotting of the Expressed Multiepitope Protein in Cell Lysates

The proteins in the COS7 cell lysates were separated on a 12% SDS-PAGE gel and transferred onto a nitrocellulose membrane by electroblotting. The membrane was blocked with 5% nonfat dry milk in TBST (TBS with 0.05% Tween-20) for 1 h at room temperature and then incubated with a polyclonal rabbit anti-DKK1 antibody (1 : 1000, Millipore) in TBST containing 0.25% bovine serum albumin overnight at 4°C. After washing three times with TBST, the membrane was incubated with horseradish peroxidase- (HRP-) conjugated goat anti-rabbit IgG (1 : 10000, ProteinTech Group, Chicago, USA) for 1 h at room temperature. After washing three times, the membrane was exposed to a SuperSignal West Pico stable peroxide solution (Pierce, ThermoScientific, Pittsburgh, PA, USA).

### 2.5. Enzyme-Linked Immunosorbent Assay (ELISA) Analysis of the Expressed Multiepitope Protein in the Cell Culture Supernatant

The protein secreted in the cell culture supernatant was detected with ELISA. Briefly, 96-well plates were coated with a goat anti-human DKK1 antibody (200 ng/mL, R&D Systems, Minneapolis, MN, USA) overnight at 4°C. The cell culture supernatant (200 *μ*L) was added to the wells and was incubated overnight at 4°C. After incubating with 100 *μ*L of a goat anti-human DKK1 antibody (50 ng/mL, R&D Systems) for 2 h at 37°C, 100 *μ*L of a working dilution of Streptavidin-HRP was added to each well. Color was developed by the addition of a substrate solution of orthophenylene diamine (OPD) for 20 min at 37°C. The reaction was stopped by the addition of 50 *μ*L of 2 M H_2_SO_4_. The absorbance was read at 450 nm by a microplate reader (Thermomax Technologies).

### 2.6. Immunohistochemical Analysis of the Multiepitope Protein* In Vivo*

BALB/c mice were injected intramuscularly with 100 *μ*g of plasmid pCMV-DP or empty vector pCMV. Seven days later, the injected muscles were surgically removed and frozen sections were prepared. The sections were dried for 45 min at room temperature, fixed with anhydrous acetone, and then incubated with 0.05% H_2_O_2_ for 20 min to quench the endogenous peroxidase. After blocking with 5% horse serum, the sections were incubated with a goat anti-human DKK1 antibody (5 *μ*g/mL, R&D Systems) overnight at 4°C. The next day, the sections were incubated with a HRP-conjugated donkey anti-goat IgG (1 : 1000, ProteinTech Group) for 1 h at room temperature. The positive signals were detected with 3, 3′ diaminobenzidine (DAB, R&D Systems).

### 2.7. Immunization and Serum Collection

For DNA immunization experiments, 6-week-old female BALB/c mice (*n* = 6) were injected intramuscularly (i.m.) with plasmid pCMV-DP three times at weeks 0, 2, and 4. Mice (*n* = 6) immunized with empty vector pCMV served as negative controls. Intramuscular injection of plasmid DNA followed by electroporation (DNA + EP) was performed as previously described [21]. Briefly, 100 *μ*g of plasmid pCMV-DP was injected intramuscularly in one tibialis anterior muscle using a 27-gauge needle. Immediately after the injection, electroporation with 6 electric pulses was applied through a pair of silver electrodes spaced 3 mm apart covering the i.m. injection site. The electric pulses were 50 ms in duration and 1 s apart at a voltage of 60 V (i.e., 200 V/cm). From the first injection, mouse serum samples were collected at 2-week intervals and stored at −80°C.

### 2.8. Antibody Assay

The antibodies against human DKK1 were evaluated using ELISA. Briefly, 96-well microtiter plates were coated with 100 *μ*L of recombinant human DKK1 proteins (200 ng/mL, R&D Systems) and incubated overnight at 4°C. After washing three times with PBST (PBS with 0.05% Tween-20), the plates were blocked with 200 *μ*L of 1% bovine serum albumin (BSA) in PBST for 2 h at 37°C. After washing, 100 *μ*L of diluted serum (1 : 200) was added to each well. Then, the plates were incubated for 2 h at 37°C and washed five times with PBST. HRP-conjugated goat anti-mouse IgG secondary antibodies (100 *μ*L) (1 : 10000, ProteinTech Group) were added to each well followed by incubation for 1 h at 37°C. Next, the plates were washed five times with PBST. After adding the substrate solution and stop solution, the absorbance was read at 450 nm by a microplate reader (Thermomax Technologies). The end-point titer of antibody was determined in the same way. The serum samples were serially diluted from 1 : 200 to 1 : 12800.

### 2.9. Preparation and Evaluation of Collagen-Induced Arthritis Mouse Model

DBA/1 mice (*n* = 6) were immunized with plasmid pCMV-DP three times at 2-week intervals as described above. Control mice (*n* = 6) were immunized with an equal amount of empty vector pCMV. One week after the final immunization, all mice were injected intradermally at the base of the tail with 100 *μ*g of bovine C II emulsified with complete Freud's adjuvant (CFA) containing 4 mg/mL of heat-killed* Mycobacterium tuberculosis*. On day 21, the animals were given a booster injection with 100 *μ*g of bovine C II dissolved in incomplete Freud's adjuvant (IFA) [22]. Animals were observed and recorded every 3 days after disease onset. Clinical scores were assigned to evaluate the disease severity as follows: 0: no signs of arthritis; 1: swelling and/or redness of one paw or one digit; 2: two joints involved; 3: three or more joints involved; and 4: severe arthritis of the entire paws and digits. Each limb was graded independently, resulting in a maximal clinical score of 16 per affected animal [23].

### 2.10. Microcomputer Tomography (CT) Scanning

To determine the protective effects of the DNA vaccine, 40 days after the challenge with bovine C II, a micro-CT-200 system (AlokA latheta Laboratory, Japan) was employed to detect the bone mineral density (BMD) and the degree of bone erosion after the mice were anaesthetized with chloral hydrate (10%). X-ray images were analyzed by reconstructed 3D quantitative analyses using the software VGstudio MAX 2.0.

### 2.11. Histopathology

Murine hind paws were removed postmortem, stored in 10% neutral formalin, decalcified in 20% ethylenediaminetetraacetic acid (EDTA) for 6 weeks, and then dehydrated and embedded in paraffin. Sections were cut along the longitudinal axis and stained with toluidine blue (TB) as previously described [18].

### 2.12. Statistical Analysis

Data were analyzed using the software SPSS (version 16.0) and presented as the mean ± SEM. Differences between two groups of mice were compared using Student's *t*-test. A *P* value less than 0.05 was considered to be statistically significant.

## 3. Results

### 3.1. Construction of the DNA Vaccine

**Figure 1:**
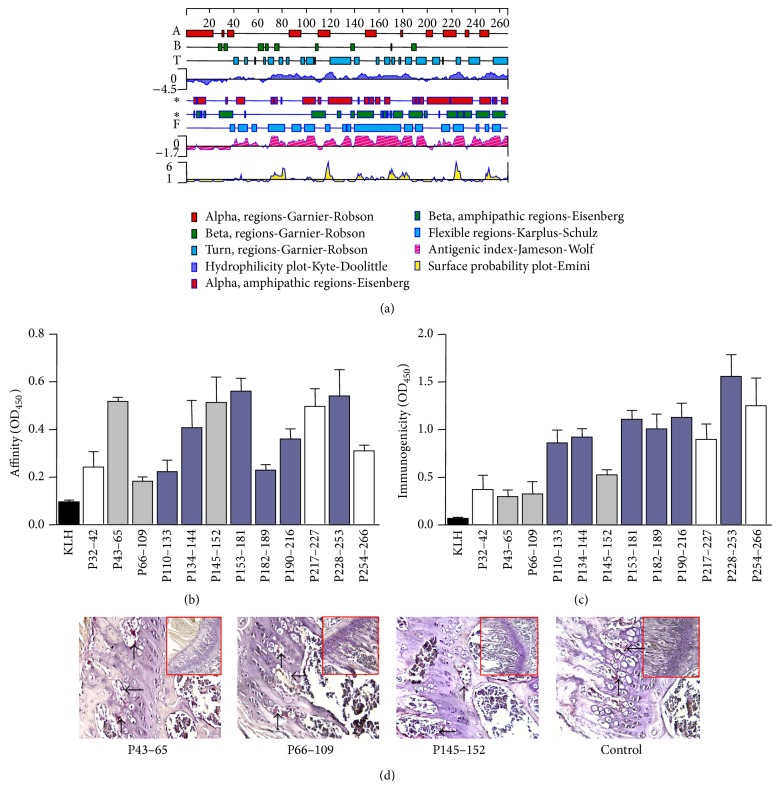
Designation of the DNA vaccine.(a) B cell epitope scanning of human DKK1 was performed with the software DNASTAR 7.1. (b) The affinity of epitopes was measured by indirect ELISA. (c) The separated epitopes were immunized BALB/c mice and the immunogenicity of epitopes was measured by sandwich ELISA. (d) The separated epitopes were injected to BALB/c mice for seven days. TRAP staining was performed to identify the mature osteoclasts. Magnification: 200x; data are expressed as the mean ± SEM.

**Figure 2:**
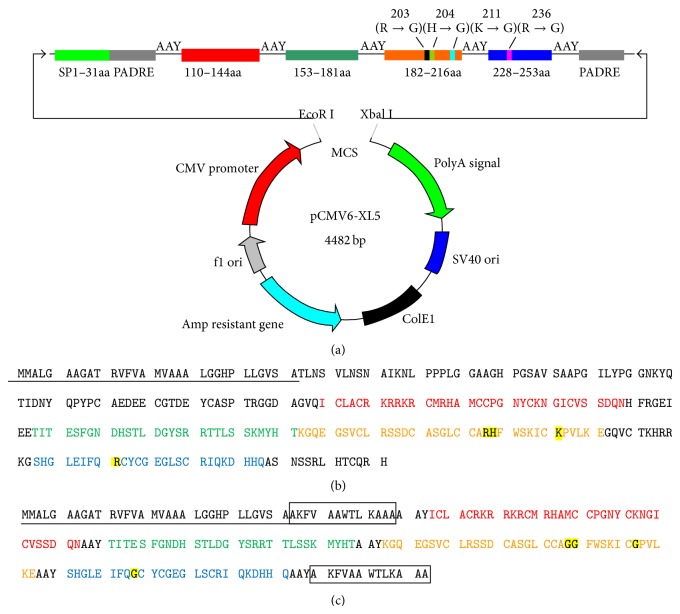
Construction of the DNA vaccine. (a) The maps of recombinant DKK1 DNA vaccine. (b) The amino acid sequence of human DKK1. (c) The recombinant amino acid sequence of DKK1 DNA vaccine. Red line, 110–144aa; green line, 153–181aa; orange line, 182–216aa; blue line, 228–253aa; black line, signal peptide; box, PADRE.

According to the analysis of the potential B cell epitopes in human DKK1 by the epitope prediction software DNASTAR 7.1, a panel of twelve peptides fragments was synthesized (Figure 1(a)). In addition, to select the high affinity and immunogenicity of synthesis peptides, the titers of peptides were determined and the peptides of grey-blue columns were candidates to design DNA vaccine (Figures 1(b)-1(c)). Furthermore, to decrease the pathological functions of DKK1 in peptides, the osteoclast-forming assay was performed. Compared with controls, no significant osteoclastogenesis was observed in peptides-treatment group (Figure 1(d)). The synthetic nucleotide sequence with muted four amino acids encoding the DNA vaccine was cloned in the eukaryotic expression vector pCMV6-XL5 (Figure 2).

### 3.2. Expression of the Multiepitope Chimera Gene* In Vitro* and* In Vivo*

**Figure 3:**
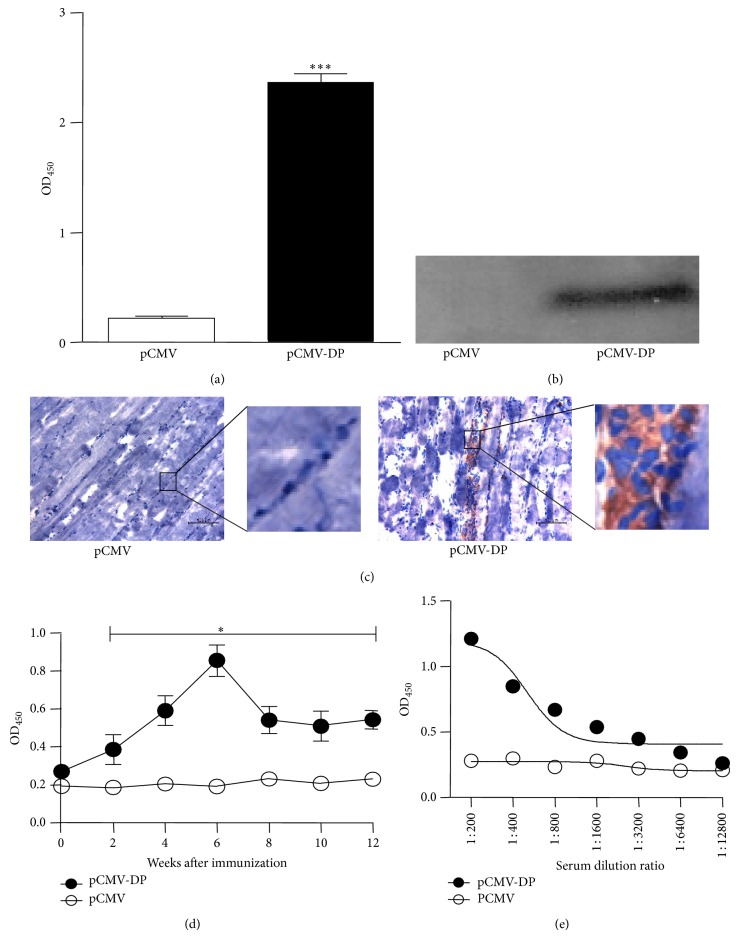
Expression and immunogenicity of the DNA vaccine. (a–c) Expression of the multiepitope protein* in vitro* and* in vivo *were determined in cell culture supernatants by ELISA, cell lysates by Western blotting, and the muscles of mice by immunohistochemical analysis. (d-e) The DNA vaccine induced a specific IgG antibody against human DKK1. The titer and the end-point titer of the specific antibody were tested by ELISA. Bars indicate 100 *μ*m. Data are expressed as the mean ± SEM, ^*^ *P* < 0.05, ^***^ *P* < 0.001.

The expression of the multiepitope DNA vaccine* in vitro* was evaluated in COS7 cells. ELISA showed that the multiepitope DNA vaccine recombinant protein of DKK1 was secreted abundantly and was recognized by its polyclonal antibodies (Figure 3(a)). In addition, Western blotting also showed that COS7 cells transfected with plasmid pCMV-DP highly expressed the recombinant protein (Figure 3(b)). To assess the expression of the target protein* in vivo*, the injected muscles of mice were further stained with an anti-DKK1 antibody. The expression of recombinant protein in the DKK1 DNA vaccine was much higher in the muscles at the injected site than in the control group (Figure 3(c)). Due to these observations, we concluded that the multiepitope gene was expressed in eukaryotes both* in vitro* and* in vivo*.

### 3.3. The DNA Vaccine Induced a Specific Antibody

A specific anti-human DKK1 antibody was identified in BALB/c mice immunized with plasmid pCMV-DP. The serum IgG titer began to increase as early as 4 weeks after the primary immunization and reached its peak at 6 weeks (Figure 3(d)). The end-point titer of the specific antibody was also determined to evaluate its neutralizing effects* in vitro* (Figure 3(e)). Although the end-point titer of anti-DKK1 was only 1 : 1600, the specific antibody existed persistently in the serum up to 6 months after immunization.

### 3.4. Induction of Arthritis in DBA/1 Mice

**Figure 4:**
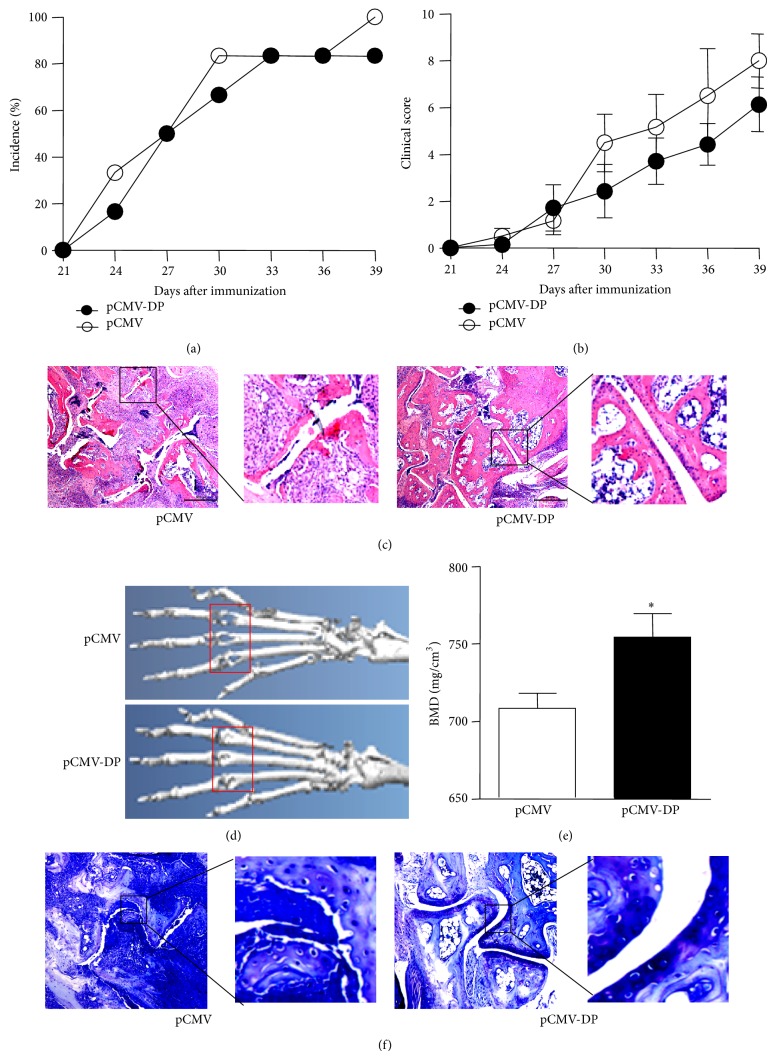
The DNA vaccine attenuated bone erosion in CIA mice. Mice were injected intradermally with bovine type II collagen to induce arthritis (*n* = 6/group). (a-b) The incidence of arthritis and clinical score were recorded until day 39. (c–f) Representative joints on the day of sacrifice were analyzed with HE staining, Micro-CT images, BMD analysis, and TB staining images. Bars indicate 500 *μ*m and 200 *μ*m. Data are expressed as the mean ± SEM. ^*^ *P* < 0.05.

DBA/1 mice were injected with bovine C II twice at 3-week intervals after immunization. The signs of arthritis appeared around day 24 after injection with bovine C II and continued to develop at later time points. The disease incidence was 83% in prevaccinated mice and 100% in the positive control mice on day 39. Paw erythema and swelling also became more severe with time in control mice than the DKK1 vaccine group (Figures 4(a) and 4(b)). In addition, the infiltration of inflammatory cells was reduced in the vaccine-immunized mice compared with the controls (Figure 4(c)).

### 3.5. The DNA Vaccine Attenuated Bone Erosion in CIA Mice

We further evaluated the amelioration of arthritic bone, and the joint structure was well conserved in the vaccinated mice compared with the positive control mice as demonstrated by microcomputer tomography (CT) scanning (Figure 4(d)). The total bone mineral density (BMD) of the mice in the vaccinated groups was significantly higher than that in the positive control group (Figure 4(e)). The results of TB staining showed that the degree of overall destruction of cartilage was reduced markedly in the arthritic paws of vaccinated mice compared with the positive controls (Figure 4(f)). The results of micro-CT scanning and TB staining demonstrated that the DNA vaccine attenuated bone erosion in CIA mice.

## 4. Discussion

DNA vaccines, also termed nucleic acid vaccines or gene vaccines, were first described in the 1990s when Wolff found that DNA could be taken up and expressed by mouse skeletal muscle cells* in vivo* [24]. It is generally thought that the gene of interest in a eukaryotic expression vector is translated into antigen protein using the cellular expression system of the host. Subsequently, the antigen protein stimulates the host to generate an immune response. Compared with traditional vaccines, the greatest advantage of DNA vaccines is their ability to elicit both humoral immunity and cellular immunity. Simultaneously, they are simple to prepare, easy to deliver, and relatively safe to apply. Therefore, DNA vaccination is a promising new technology for the prevention and therapy of diseases, such as infectious diseases, autoimmune diseases, and cancers.

In this study, we constructed a DNA vaccine targeting human DKK1, a major contributor to bone loss in RA. We expected to use this vaccine to inhibit bone erosion in a RA mouse model through the blockade of the biological activity of DKK1. We have adopted optimization strategies to improve the antigen expression and the immunogenicity of the DNA vaccine, which are discussed below.

### 4.1. Construction Strategies for the DNA Vaccine

To produce specific antibodies, B cell epitopes of DKK1 were the first choice to construct the DNA vaccine. To increase the titer of specific antibodies, we combined the predictions of the software DNASTAR 7.1 and the affinity and immunogenicity assays. To reduce their potential bone resorption of natural DKK1, the synthesis epitopes were injected to BALB/c and no significant osteoclastogenesis was observed. These data demonstrated that these separated epitopes were safe as a vaccine utilized* in vivo*. To increase the immunogenicity of the DNA vaccine as expressed in the host, the key binding sites of DKK1 (R203, H204, K211, and R236) to LRP5/6 and Kremen were mutated. At the same time, the mutated protein expressed* in vivo* enhanced the immunogenicity of the vaccine [20]. To facilitate the epitope processing, the four selected epitopes were separated from one another with AAY spacers [25]. AAYs expressed inside the selected epitopes were easily recognized by proteasomes, and the full-length protein was more easily processed and presented* in vivo*. DKK1 is expressed naturally in the body. To further increase the immunogenicity of the recombinant DNA vaccine, the pan DR T helper epitope (PADRE) was added to enhance antibody responses [26]. Finally, the signal peptide for human DKK1 was added at the N-terminus of the DNA vaccine to facilitate the extracellular secretion of the protein antigen.

### 4.2. Immunization Strategies

The DNA vaccine delivery method is one of the most important factors that affect immunization efficiency. The methods for DNA vaccine delivery currently used include intramuscular, intraperitoneal, intravenous, intradermal, subcutaneous injection, and others. The delivery method for the plasmid directly affects the uptake of the gene and further affects the expression of the target protein. Intramuscular injection is the earliest and simplest method for DNA delivery. However, the majority of the administered plasmid DNA was blocked by perimysia of the skeletal muscles, where most of the DNA was degraded by DNase I in the plasma. Less than 1% of the DNA actually entered the nucleus [27]. Recently, electroporation has been used for DNA vaccine delivery and has proven to be a better DNA delivery method. In this study, intramuscular injection of plasmid DNA followed by electroporation (DNA + EP) was employed. The DC electric current disrupts cell membranes for a short time, allowing the plasmid DNA to cross the membranes into the nucleus [28]. In addition, the electric current causes cell destruction and a local inflammatory reaction. A large number of inflammatory cells, including macrophages and other antigen-presenting cells, infiltrated the injected muscles, which enhanced the efficiency of antigen presentation. In our study, the highly specific IgG antibodies in serum were induced as early as 4 weeks, which also demonstrated the efficiency of the electroporation of the DNA vaccine.

## 5. Conclusions

DKK1, a negative regulator of the canonical Wnt signaling pathway, plays an important role in RA bone destruction. Inhibition of its biological functions can attenuate bone destruction in RA [9]. Our results showed that the recombinant human DKK1 multiepitope DNA vaccine induced specific antibodies that effectively neutralized the biological activities of DKK1 and attenuated bone destruction in CIA mice. This study may provide a potential therapy for RA and other bone loss diseases.
